# In-Vivo Evaluation of Peripheral Refraction Changes with Single Vision and Multifocal Soft Contact Lenses

**Published:** 2018

**Authors:** Jie Shen, Frank Spors, Dorcas Tsang, Lance E. McNaughton, Donald J. Egan

**Affiliations:** 1 Western University of Health Sciences, College of Optometry, Pomona, CA, USA; 2 University of Pikeville, Kentucky College of Optometry, Pikeville, KY, USA

**Keywords:** Contact Lens, Peripheral Refraction, Myopia, Optics, Wavefront Aberrations, Multifocal, Aberrometer

## Abstract

This study investigated in-vivo changes of peripheral refraction with commercially available single vision and multifocal soft contact lenses, utilizing different designs and various corrective power values. Starting at the fovea, wave-front aberrations were measured up to 30^o^ nasal retinal eccentricity, in 10^o^ increments, using a commercially available Shack-Hartmann aberrometer. Three different types of contact lenses were fitted in an adult subject’s right eye: Acuvue Oasys Single Vision (ASV), Proclear Multifocal D with 2.50 diopters (D) add power (PMD), and ArtMost SoftOK (SOK). Each lens type was fitted in corrective power values of -2.00 D, -4.00 D, and -6.00 D. Refractive errors were computed in power vector notation: The spherical equivalent (M), the Cartesian Jackson-Cross-Cylinder (J_0_), and the oblique Jackson Cross Cylinder (J_45_) from measured second order Zernike terms. Acuvue Oasys Single Vision lenses produced a slight myopic shift at 30^o^ retinal periphery (-0.32 D ± 0.05) without significant differences between the various lens power values. Proclear Multifocal D lenses did not create clinically significant myopic shifts of at least -0.25 D. All SOK lenses produced clinically significant relative myopic shifts at both 20^o^ (-0.61 D ± 0.08) and 30^o^ (-1.42 D ± 0.15) without significant differences between the various lens power values. For all lens types and power values, off-axis astigmatism J_0_ was increased peripherally and reached clinical significance beyond 20^o^ retinal eccentricity. The increased amount of off-axis astigmatism J_0_ did not show a significant difference for the same type of lenses with different dioptric power. However, at 30^o^ retinal eccentricity, SOK lenses produced significantly higher amounts of off-axis astigmatism J_0_, compared with ASV and PMD lenses (SOK versus ASV versus PMD: -1.67 D ± 0.09, -0.81 D ± 0.07, and -0.72 D ± 0.15). Both ASV and SOK lenses showed no clinically significant differences in the amount of introduced astigmatic retinal image blur, with various lens power values. Proclear Multifocal D lenses showed a systematic increase of astigmatic retinal image blur with an increase of add power. At 30^o^ retinal eccentricity, -6.00 D SOK lenses introduced 0.73 D astigmatic retinal image blur, while PMD and ASV lenses introduced 0.54 D and 0.37 D, respectively. In conclusion, relative peripheral refractions, measured in-vivo, were independent of the contact lenses central corrective power. The SOK contact lenses demonstrated a stronger capability in rendering relative peripheral myopic defocus into far periphery, compared to the other lens designs used in this study. This was accompanied by higher amounts of introduced astigmatic retinal image blur.

## Introduction

Myopia is a common type of refractive error, which can be differentiated to axial myopia and refractive myopia. Axial myopia is primarily caused by an axial elongation of the eyeball, which exceeds the refractive power of the eye’s optical system, therefore, the image is formed in front of the retina. There are higher risks of retinal detachment, glaucoma, possible blindness, and other ocular pathologies in the presence of axial myopia [1]. This poses economical as well as health care burdens to myopic individuals. In terms of costs to the society, there is an estimated $4.6 billion dollars annual expenditure related to myopia [2]. Worldwide, the prevalence of myopia was increased in the recent years with some East Asian countries reporting prevalence rates of up to 80% [3, 4].

A robust body of scientific literature suggested that onset and progression of myopia are related to the direction of retinal defocus. A hyperopic defocus will cause continuous ocular growth to compensate for the blurred retinal image, while, a myopic retinal defocus acts as a stop signal [5-7]. Animal studies [8, 9], which include mammalian models [10, 11], as well as primates [12, 13], have confirmed this theory. Moreover, this theory applies to both the foveal portion of the eye, as well as the retinal periphery [14, 15]. The human fovea occupies only 1% of the central retinal area, while the remaining 99% represents the retinal periphery [16]. Therefore, it is reasonable to assume that a peripheral retinal visual stimulus has the ability to substantially influence the progression of myopia.

Many studies have reported that emmetropic and hyperopic eyes tend to have relative myopic retinal peripheries, while myopic eyes tend to have relative hyperopic retinal peripheries, at least along their horizontal visual fields [17-19]. It seems plausible that a myopic eye will continue to experience myopic progression as long as its retinal periphery receives a hyperopic defocus. This hypothesis has been confirmed by several scientific studies [20, 21].

Orthokeratology is one of the most effective optical interventions for slowing the progression of myopia [22, 23]. With this treatment option, changes in corneal topography ultimately result in a myopization of the retinal periphery and, therefore, contributes to a robust myopia control effect [24]. However, potential issues may limit the widespread use of orthokeratology, such as, discomfort while wearing rigid contact lenses, relative complicated lens fitting and lens care procedures, treatment costs, and a potentially increased risk of corneal infections [25, 26]. Multifocal soft contact lenses can be specifically designed to provide optics, which are comparable to those of corneas during orthokeratology. Various studies have demonstrated a myopia control effect with these lenses, which is comparable to orthokeratology [27, 28].

Multifocal soft contact lenses with center distance designs, which are developed to be used in presbyopic patients, may also be used off label for myopia control [28]. Several studies have investigated the in-vivo optics of some of these stock lens designs when used in the context of myopia control, especially the amount of induced peripheral defocus [29-31].

In the recent years, new multifocal contact lens designs were specifically developed for myopia progression control [32]. Therefore, a continued interest in in-vivo optical performance of these contact lenses, as well as a comparison with established lens designs exists.

The purpose of this study was to investigate in-vivo changes of peripheral refraction with commercially available single vision and multifocal soft contact lenses, utilizing different designs and various corrective power values.

## MATERIALS AND METHODS

**Contact Lenses**

The researchers fitted three different types of contact lenses for the participant: Acuvue Oasys® single vision (ASV), Proclear® Multifocal D with +2.50 diopter (D) add power (PMD), and ArtMost SoftOK® (SOK). Each lens type was assessed with corrective power values of -2.00 D, -4.00 D, and -6.00 D. The PMD as well as the SOK contact lenses possess multifocal optics. While the PMD lens design was developed for presbyopia, SOK was specifically designed to mimic the optical performance established in orthokeratology.

**Instrumentation and Set-up**

Using a commercially available Complete Ophthalmic Analysis System (COAS) Shack-Hartmann aberrometer (AMO Wavefront Sciences, Inc., Albuquerque, New Mexico), the researchers measured wavefront aberrations with and without soft contact lenses in an adult subjects’ right eye, which was used as a stable test case (male, 41 years old, refractive error: -6.00 D sphere and -0.50 D cylinder with axis at 173^o^, no ocular pathologies). This interventional study was approved by the Institutional Review Board of Western University of Health Sciences and informed consent was obtained from the subject. Starting at the patient’s fovea, the researchers took measurements in 10^o^ increments, extending out to 30^o^ nasal retinal eccentricity. Three measurements were taken at each gaze position. The instrument was realigned to the measured eye before each measurement.

**Data Analysis**

The researchers computed relative power vector values of defocus M, with-the-rule (WTR) and against-the-rule (ATR) astigmatism J_0_ (the Cartesian Jackson-Cross-Cylinder), and oblique astigmatism J_45_ (the oblique Jackson Cross Cylinder), by using the following set of equations from second order Zernike terms: [33]

$M=\frac{-4\sqrt[{2}]{3}}{r^{2}}C_{2}^{0}$

$J_{0}=\frac{-4\sqrt[{2}]{3}}{r^{2}}C_{2}^{2}$

$J_{45}=\frac{-2\sqrt[{2}]{6}}{r^{2}}C_{2}^{-2}$

Where Cs are Zernike coefficients for defocus (C_2_^0^), WTR/ATR astigmatism (C_2_^2^) and oblique astigmatism (C_2_^-2^), M is the spherical equivalent, and *r* is the pupil radius.

One-sample Kolmogorov-Smirnov test at 5% significance level was applied to the data, as statistical analysis, using Matlab (MathWorks, Inc., Natick, MA). Figures presented in this paper were also generated using the Matlab program. For further interpretation, the researchers considered a change of 0.25 D in any of the power vectors as clinically significant.

In addition, this study analyzed relative astigmatic retinal image blur, by comparing the root mean square value of the combined power vectors J_0_ and J_45_ of each lens design, using the following equation: [34]

## RESULTS

**Defocus M**

**Figure 1:**
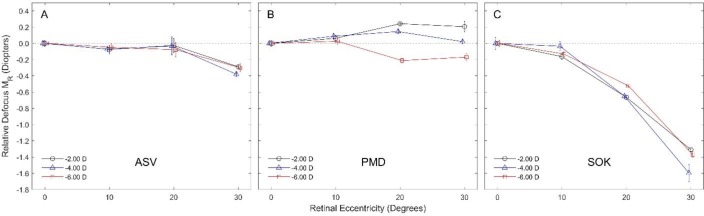
Relative defocus MR with three diﬀerent contact lens types, each having three diﬀerent power values, at various degrees of nasal retinal eccentricity.

Acuvue Oasys® single vision lenses produced a slight, yet clinically significant relative myopic defocus at 30^o^ retinal eccentricity (-0.32 D ± 0.05) without a significant difference between the various lens power values (Figure 1A). Furthermore, PMD lenses did not create clinically signiﬁcant changes in defocus across the measured nasal retinal field (Figure 1B) and SOK lenses produced a pronounced and clinically signiﬁcant relative myopic defocus at 20^o^ (-0.61 D ± 0.08) and more so 30^o^ (-1.42 D ± 0.15) retinal eccentricity, without significant diﬀerences between the various lens power values (Figure 1C).

(M_R_: Peripheral M – Central M; D: diopter; ASV: Acuvue Oasys® single vision contact lens; PMD: Proclear Multifocal D with 2.50 diopter add power contact lens; SOK: ArtMost SoftOK contact lens).

**With-the-Rule and Against-the-rule Astigmatism J**_0_

**Figure 2:**
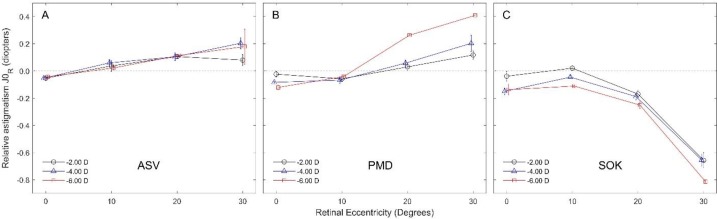
Relative WTR/ATR astigmatism.

Acuvue Oasys® single vision lenses did not signiﬁcantly impact astigmatism J_0_ across the measured nasal retinal ﬁeld, although with increasing peripheral field angle, the WTR component of this power vector increased (Figure 2A). Furthermore, PMD lenses did not signiﬁcantly impact J_0_ astigmatism, except the -6.00 D lens, which created a signiﬁcant increase of the WTR component of this power vector at 20^o^ (+0.26 D) and 30^o^ (+0.41 D) retinal eccentricity (Figure 2B). The SOK lenses signiﬁcantly impacted J_0_ astigmatism at 30^o^ retinal eccentricity (-0.71 D ± 0.09) for all power values, and in contrast to the other lenses, produced ATR astigmatism. In addition, the -6.00 D lens signiﬁcantly changed J_0_ at 20^o^ retinal eccentricity (-0.25 D) (Figure 2C).

**Oblique Astigmatism J**_45_

**Figure 3:**
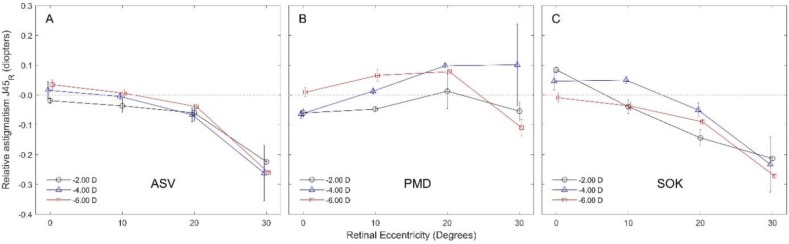
Relative oblique astigmatism J_45_ with three diﬀerent contact lens types, each having three diﬀerent power values, at various degrees of nasal retinal eccentricity.

Acuvue Oasys® single vision lenses clinically signiﬁcantly impacted astigmatism J_45_ at 30^o^ retinal ﬁeld for the -4.00 D and -6.00 D power values, which increased J_45_ by -0.26 D (Figure 3A). Furthermore, PMD lenses did not signiﬁcantly impact astigmatism J_45_ across the measured nasal retinal ﬁeld (Figure 3B) and SOK lenses had a clinically signiﬁcant impact on astigmatism J_45_ at 30^o^ retinal ﬁeld for the -6.00 D power value, which increased J_45_ by -0.27 D (Figure 3C).

(WTR: with-the-rule; ATR: against-the-rule D: diopter; ASV: Acuvue Oasys® single vision contact lens; PMD: Proclear Multifocal D with 2.50 diopter add power contact lens; SOK: ArtMost SoftOK contact lens).

(J_45_: the oblique Jackson Cross Cylinder; D: diopter; ASV: Acuvue Oasys® single vision contact lens; PMD: Proclear Multifocal D with 2.50 diopter add power contact lens; SOK: ArtMost SoftOK contact lens).

**Figure 4:**
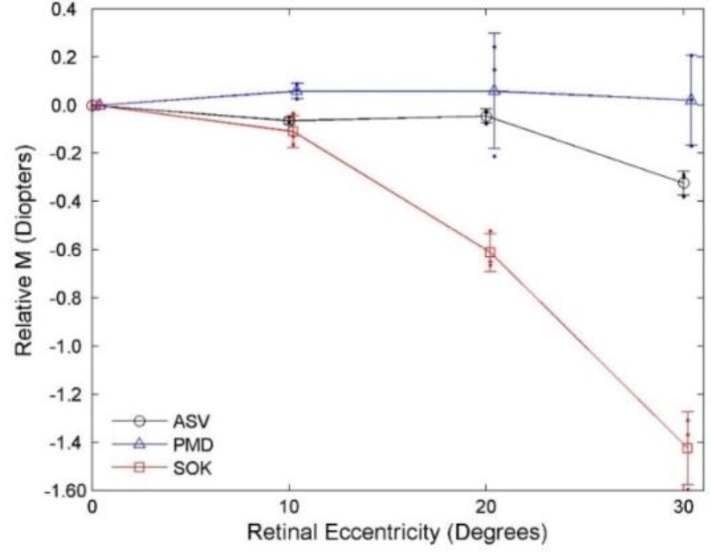
Direct comparison of relative change in peripheral refraction with three diﬀerent contact lens types at various degrees of nasal retinal ccentricity. Each curve indicates an average relative value of three utilized powers per lens (-2.00 D, -4.00 D, -6.00 D) for power vectors M.

(ASV: Acuvue Oasys® single vision contact lens; PMD: Proclear Multifocal D with 2.50 diopter add power contact lens; SOK: ArtMost SoftOK contact lens).

**Direct Comparison of Average Changes in Peripheral Defocus M**

For an easier visual comparison of the average changes in M across the nasal retinal field, the researchers plotted the average relative mean changes of the three utilized power values per lens (-2.00 D, -4.00 D, -6.00 D) across the nasal visual field (Figure 4).

**Direct Comparison of Astigmatic Retinal Image Blur**

**Figure 5:**
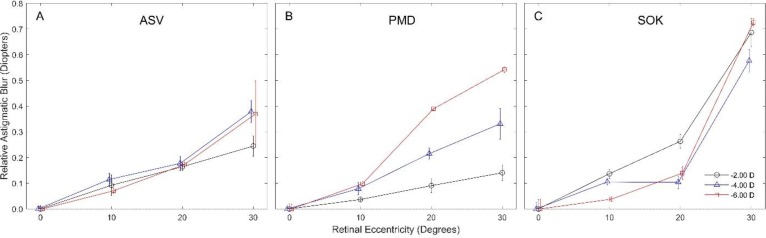
Relative astigmatic retinal image blur with three diﬀerent contact lens types at various degrees of nasal retinal eccentricity. Each curve indicates the root mean square value of the combined power vectors J_0_ and J_45_.

When analyzing relative astigmatic retinal image blur, the researchers found a higher effect for SOK lenses compared to ASV and PMD lenses, especially at the 30^o^ nasal retinal (Figure 5).

(J_0_: Cartesian Jackson-Cross-Cylinder, J45: the oblique Jackson Cross Cylinder, D: diopter; ASV: Acuvue Oasys® single vision contact lens; PMD: Proclear Multifocal D with 2.50 diopter add power contact lens; SOK: ArtMost SoftOK contact lens.)

## DISCUSSION

Measurements on peripheral refraction using the Complete Ophthalmic Analysis System (COAS) has been validated in a previous study, especially when measuring eyes fitted with multifocal contact lenses [31]. For a particular lens type, the results indicate that changes of peripheral refraction in the nasal retina were independent of central corrective power values of individual lenses. ArtMost SoftOK lenses demonstrated the strongest capability in producing relative peripheral myopic defocus at the outer nasal retinal periphery. According to the hypothesis that peripheral myopic defocus is a protective mechanism for further development of myopia, it is reasonable to assume that the SOK lens will have a strong potential for inhibiting myopia progression. 

The current results also suggested that the SOK lens design induced a clinically significant amount of peripheral ATR astigmatism. The researchers found astigmatic retinal image blur at the 30^o^ nasal retinal periphery to be higher with SOK lenses compared to ASV and PMD lenses. Although astigmatic blur certainly influences retinal image quality, animal studies have shown that defocus has a stronger effect on eye growth [35, 36].

The single eye approach used in this study was successful as it provides a stable case for fitting contact lenses with different designs and power values. The current results rendered information to the field, to better understand the power profiles in the peripheral visual field after wearing the three tested soft contact lenses and looked at the differences of peripheral refraction introduced by the same design with different power values. However, the temporal visual field was not measured due to the limitation of the device to access the full horizontal visual field. More lens power values could be chosen from each type of contact lenses to better evaluate the changes in their peripheral refraction. 

More studies are needed to evaluate in-vivo changes in peripheral refraction with a wider variety of soft contact lens designs in the horizontal and vertical retinal fields. In addition, interventional studies could identify a specific amount of change in peripheral retinal refraction, required for efficiently controlling myopia progression in children.

## CONCLUSIONS

In this study, relative peripheral refractions, measured in-vivo, were independent of the contact lenses central corrective power. The SOK contact lenses demonstrated a stronger capability in rendering relative peripheral myopic defocus to far periphery, compared to the other lens designs used in this study. This was accompanied by higher amounts of introduced astigmatic retinal image blur.
